# Diagnostic validity of specific immunoglobulin E levels to alpha-gal in alpha-gal syndrome: a cross-sectional analysis

**DOI:** 10.1186/s13223-023-00856-6

**Published:** 2023-11-30

**Authors:** Adrián Germán-Sánchez, Ana Alonso-Llamazares, Fernando García-González, Bakai Matala-Ahmed, Ceny Solani Melgar-Reyes, Ignacio Antepara-Ercoreca

**Affiliations:** 1 Allergy Department, Basurto University Hospital, Bilbao, Spain; 2 Allergy Department, Castellon University General Hospital, Avda/ Benicassim, 128, Castelló de la Plana 12004 (Castelló, Castellon, Spain

**Keywords:** Diagnostic validity, Specific immunoglobulin E, Alpha-gal syndrome, Food allergy

## Abstract

**Background:**

The diagnosis of Alpha-gal Syndrome (AGS) is based on the presence of symptoms after being exposed to potential sources of alpha-gal together with values ​​of specific IgE (sIgE) to alpha-gal ≥ 0.1 kUA/L or ≥ 0.35 kUA/L. The aim of this study was to evaluate the diagnostic validity of sIgE levels to alpha-gal ≥ 0.1 kUA/L for identifying AGS.

**Methods:**

This was a cross-sectional analysis of adult patients with available data on sIgE levels to alpha-gal, classified into two groups according to the presence (Group 1) or absence (Group 2) of symptoms after being exposed to potential sources of alpha-gal. Values of sIgE to alpha-gal ≥ 0.1 kUA/l were considered a positive result. A descriptive analysis of internal and external validity parameters was performed in the entire population and adjusted by sex.

**Results:**

The study included 33 individuals in Group 1 and 65 in Group 2, with a mean age of around 47 years. The analysis of internal validity parameters revealed a high sensitivity, specificity, and positive probability ratio, with higher sensitivity in men and higher specificity in women. The analysis of external validity parameters showed a high negative predictive value and global value in all populations and both sexes. However, the positive predictive value was relatively high in men, but low in women.

**Conclusions:**

Our results suggest that sIgE levels ≥ 0.1 kUA/L may be a useful tool for the diagnosis of AGS, although other factors and diagnostic techniques should also be considered.

## Introduction

Alpha-gal Syndrome (AGS) is an allergy related to tick bites. Ticks induce sensitization to the oligosaccharide galactose-alpha-1,3-galactose (alpha-gal), a glycan of nonprimate mammals that is homologous to the B-group blood antigen [1]. Different tick species have been associated with AGS worldwide, such as *Amblyoma americanum, Ixodes ricinus*, *Ixodes holocyclus*, or *Haemaphysalis longicornis* [2]. Once a patient is sensitized to alpha-gal, the ingestion of mammalian meat or viscera (e.g., kidney, liver) triggers the development of delayed allergic reactions that manifest with gastrointestinal symptoms (nausea, vomiting, or abdominal pain), urticaria, angioedema, or life-threatening anaphylaxis. These reactions are often difficult to avoid because alpha-gal is also present in a large number of products, such as dairy, medicines (e.g., cetuximab), food additives (e.g., gelatin), or other products from mammalian sources [3].

The diagnosis of AGS is based on the presence of symptoms after exposure to a source of alpha-gal, together with values ​​of specific IgE (sIgE) to alpha-gal ≥ 0.1 kU/L [4] or ≥ 0.35 kU/L [5]. The data published worldwide do not show significant differences between sexes in the sIgE response to alpha-gal or in the presentation of signs and symptoms during the reaction, although they have been related to the practice of outdoor activities [6]. Other studies have associated AGS with alpha-gal sIgE levels > 2IU/mL or > 2% with respect to total IgE levels. Nevertheless, one case with a convincing history of AGS showed different values (total IgE levels of 6.0 IU/mL and sIgE to alpha-gal of 0.8 IU/mL) [7]. In addition, a systematic review evaluating the diagnostic performance of sIgE to alpha-gal in 135 patients with red meat allergy and 37 controls reported a sensitivity of 100% and a specificity of 92.3%, with a high positive predictive value (PPV) and a negative predictive value (NPV) ≤ 50% if the pretest probability of red meat allergy was less than approximately 90% [8].

The validity of a measure is calculated by evaluating the presence or absence of a result from a reference criterion using internal and external parameters. It represents the degree to which the results of a study are valid for the population that has been studied; and the degree to which these results can be extrapolated to other populations [9]. Despite the above data on sIgE levels to alpha-gal as a diagnostic tool, its validity has not been evaluated yet. Therefore, the aim of this study was to evaluate the diagnostic validity of the levels of sIgE to alpha-gal as a diagnostic tool for AGS, in a series of patients from Bilbao, Spain.

## Methods

This was a cross-sectional analysis of data from a case-control study investigating AGS in patients who attended the Allergy Department of the OSI Bilbao-Basurto hospital (North of Spain) between 2016 and 2019. The study included two groups of patients: Group 1, which consisted of patients with AGS symptoms after being exposed to potential sources of alpha-gal, and Group 2, a control group composed of patients without AGS symptoms after being exposed to these sources. Patients with AGS symptoms presented with urticaria, angioedema, or anaphylaxis after the ingestion of mammalian products, the infusion of cetuximab, or the administration of mammalian gelatins. Patients in control group presented with urticaria, angioedema or anaphylaxis without symptoms after the ingestion of products derived from mammals, regardless of the presence of atopy. Patients were included in the study if they were aged ≥ 18 years and had available data on sIgE levels to alpha-gal. All patients signed an informed consent to participate in the study, which was approved by the local ethics committee.

The following variables were collected: sex, age, history of tick bites, participation in outdoor activities, symptoms after exposure to potential sources of alpha-gal, tolerance to potential sources of alpha-gal, total IgE levels, and sIgE levels to alpha-gal determined by ImmunoCAP (Thermofisher). The sIgE test was considered positive if the levels of sIgE to alpha-gal were ≥ 0.1 kUA/L.

Patients were classified according to these criteria in the entire population and adjusted by sex. Quantitative variables were described as the mean and standard deviation (SD), whereas categorical variables were presented as frequencies and percentages. Due to the small sample size, a descriptive analysis of internal and external validity parameters was performed but a formal statistical analysis was not conducted [10].

## Results

### Characteristics of study patients

As shown in Table 1, the study included 33 patients in Group 1 and 65 controls in Group 2 with a mean age of around 47 years. Most patients in Group 1 (90.9%) were males, while in Group 2 there were more women (65.5%) than men (38.5%). In Group 1, most patients (81.8%) had a history of tick bites and all of patients (100%) regularly performed outdoor activities. Mean levels of total IgE were higher in Group 1 (495.99 kUA/L) than in Group 2 (334.10 kUA/L). Finally, the levels of sIgE to alpha-gal were ≥ 0.1 kUA/L in 98.0% of patients in Group 1, and in 10.8% of the patients in Group 2.

**Table 1:** Characteristics of study patients according to the presence or absence of AGS symptoms with potential sources of alpha-gal and their sex

	Group 1 (n = 33)	Group 2 (n = 65)
**Sociodemographic Data**		
Sex, *n(%)*		
Women Men	3 (9.1) 30 (90.9)	40 (65.5) 25 (38.5)
Age (years), *mean (SD)* Women Men	46.91 (13.24) 36.33 (6.60) 47.97 (13.27)	47.65 (14.84) 45.23 (14.36) 51.52 (14.77)
Tick bites, *n (%)* Women Men	27 (81.8) 2 (66.7) 25 (83.3)	18 (27.7) 11 (27.5) 7 (28.0)
Outdoor activities, *n (%)* Women Men	33 (100.0) 30 (100.0) 3 (100.0)	49 (75.4) 28 (70.0) 21 (84.0)
**Clinical Features**		
Symptoms with potential sources of alpha-gal, *n (%)* Women Men	33 (100.0) 3 (9.1) 30 (90.9)	0 (0.0) 0 (0.0) 0 (0.0)
Tolerance to potential sources of alpha-gal, *n (%)* Women Men	0 (0.0) 0 (0.0) 0 (0.0)	65 (100.0) 40 (65.5) 25 (38.5)
**Immunological Data**		
Total IgE (kUA/L), *mean (SD)* Women Men	495.99 (747.65) 308.73 (363.37) 514.72 (773.18)	334.10 (601.85) 244.54 (474.37) 477.39 (740.53)
sIgE to alpha-gal (kUA/L), *mean (SD)* Women Men	31.70 (36.06) 0.81(0.35) 9.29 (44.45)	0.28 (1.41) 0.03 (0.12) 0.67 (2.21)
sIgE to alpha-gal ≥ 0.1 kUA/L, *n (%)* Women Men	32 (98.0) 2 (6.1) 30 (90.9)	7 (10.8) 2 (3.1) 5 (7.7)
sIgE to alpha-gal ≥ 2.0 kUA/L, *n (%)* Women Men	26 (78.79) 0 (0.0) 26 (86.67)	2 (3.08) 0 (0.0) 2 (8.00)

*Group 1*: Patients with AGS symptoms after being exposed to potential sources of alpha-gal

*Group 2*: Patients without AGS symptoms after being exposed to potential sources of alpha-gal

AGS, Alpha-gal Syndrome; kUA/L, kilounits or antibody per liter; sIgE, specific IgE

### Diagnostic validity of sIgE values to alpha-gal

Table 2 shows the classification of patients according to their symptoms and the test results for sIgE to alpha-gal as true positives, false positives, false negatives, or true negatives in the entire population and by sex.

**Table 2:** Classification of patients according to the presence of AGS symptoms with potential sources of alpha-gal and their test results for sIgE to alpha-gal, n *(%)*

	Group 1	Group 2	Total
Population			
Positive test	32 (97.1)^a^	7 (10.8)^b^	39 (39.8)
Negative test	1 (3.0)^c^	58 (89.2)^d^	59 (60.2)
Total	33 (100.0)	65 (100.0)	98 (100.0)
**Women**			
Positive test	2 (66.7)^a^	2 (5.0)^b^	4 (9.3)
Negative test	1 (33.3)^c^	38 (95.0)^d^	39 (90.7)
Total	3 (100.0)	40 (100.0)	43 (100.0)
**Men**			
Positive test	30 (100.0)^a^	5 (20.0)^b^	35 (63.6)
Negative test	0 (0.0)^c^	20 (80.0)^d^	20 (36.4)
Total	30 (100.0)	25 (100.0)	55 (100.0)

*Group 1*: Patients with AGS symptoms after being exposed to potential sources of alpha-gal

*Group 2*: Patients without AGS symptoms after being exposed to potential sources of alpha-gal

^a^*True positive*: patients with AGS symptoms after exposure to a source of alpha-gal with values sIgE ≥ 0.1 KUA/L

^b^*False positive*: patients without AGS symptoms after exposure to a source of alpha-gal with values sIgE ≥ 0.1 KUA/L

^c^*False negative*: patients with AGS symptoms after exposure to a source of alpha-gal with values sIgE < 0.1 KUA/L

^d^*True negative*: patients without AGS symptoms after exposure to a source of alpha-gal with values sIgE < 0.1 KUA/L

AGS, Alpha-gal Syndrome; sIgE, specific IgE

Subsequently, the analysis of internal validity parameters revealed a high sensitivity (0.97), specificity (0.89), and positive probability ratio (9.00), with a higher sensitivity in men (1.00) and a higher specificity in women (0.95). In addition, the analysis of external validity parameters showed a high negative predictive value (0.98) and global value in the whole study population (0.92) and both sexes (0.91 in men and 0.93 in women). Nevertheless, the positive predictive value was relatively high in men (0.86), but low in women (0.50) (Table 3).

**Table 3:** Internal and external validity parameters of sIgE values to alpha-gal

	Population	Women	Men
Internal Validity Parameters			
Sensitivity	0.97	0.67	1.00
Specificity	0.89	0.95	0.80
False positive rate	0.11	0.05	0.20
False negative rate	0.03	0.33	0.00
Positive probability ratio	9.00	13.33	5.00
Negative probability ratio	0.03	0.35	0.00
**External Validity Parameters**			
Positive predictive value	0.82	0.50	0.86
Negative predictive value	0.98	0.97	1.00
Global value	0.92	0.93	0.91

## Discussion

Our results emphasize the validity of a diagnostic test that has not been properly evaluated to date. In this assessment, a cut-off value for sIgE to alpha-gal ≥ 0.1 kUA/L was able to diagnose 97% of patients with symptoms after exposure to potential alpha-gal sources, with good internal and external validity. In addition, the characteristics of the patients in this group, suggest a relationship between sensitization to this oligosaccharide and a history of tick bites, as previously described [2].

The most common method to confirm the diagnosis of AGS is the use of serologic testing for alpha-gal sIgE levels ≥ 0.1 kUA/L [11]. Moreover, the classic features of AGS include the presence of positive sIgE levels to alpha-gal [7]. Other diagnostic methods are available, such as skin testing with commercial extracts of mammalian meat, but they have lower sensitivity, or prick-to-prick testing with cooked meats, or skin prick and/or intradermal testing with cetuximab or gelatin, which present a strong correlation with alpha-gal sIgE levels [11]. In addition, it should be taken into account that patients with anaphylaxis due to AGS and indolent systemic mastocytosis can present with lower values of sIgE to alpha-gal [12]. However, none of the patients in our study presented mastocytosis and the determination of sIgE levels to alpha-gal allowed us to diagnose AGS with a good validity of the results.

Other researchers have proposed an integrative diagnostic methodology that combines medical history with anti-alpha-gal IgE titers in a machine learning algorithm because it is possible that the cutoff value of sIgE that reveals clinical reactivity might not be well determined since AGS can be highly influenced by the presence of cofactors and the delayed nature of the reaction [13]. Although the presence of cofactors was not taken into account in the analysis, 11 individuals from Group 1 (9 men and 2 women) had presented them in the initial reaction, highlighting their role in the diagnosis.

Current strategies for diagnosing AGS explained that the relevance of positive testing to alpha-gal sIgE levels with a cut-off value ≥ 0.35 kU/L remains unclear because it is not able to predict the severity of the reactions [14]. Other authors have shown that the cut-off for sIgE to alpha-gal associated with a positive predictive value of > 95% probability of presenting meat allergy was 2.00 kU/L [15]. In our study, the results revealed that a cut-off for sIgE to alpha-gal ≥ 0.1 kUA/L was able to diagnose AGS in all patients with a positive predictive value of 82%, although the performance of a challenge-proven meat allergy test could modify the diagnosis of some patients. However, if the cut-off point applied was ≥ 2 kUA/L as in the study of Mabelante et al. [15], the positive predictive value of specific IgE to alpha-gal would be 93%.

The predictive test cut-offs in food allergens offers guidance in determining successful oral tolerance [16]. The validation of sIgE for the diagnosis of egg allergy in children has also been reported, with a sensitivity of 0.91 and positive predictive value of 0.94 to sIgE levels for egg white ≥ 0.35 kUA/L [17]. In our study, a cut-off value of ≥ 0.1 kUA/L also showed high sensitivity and specificity (0.97 and 0.89 respectively), and a relatively high positive predictive value (0.82), with sensitivity being higher in men and specificity being higher in women. In addition, a previous study on sIgE to alpha-gal to diagnose red meat allergy showed a sensitivity of 1.00 and specificity of 0.92, which were higher than in our population, but had a lower positive predictive value (≤ 50%). Futhermore, as in our study, the proportion of men with AGS symptoms was higher than women, since 55.6% of the subjects were male, although the authors did not perform an analysis adjusted by sex as we did [8].

The analysis of internal validity parameters showed a high positive probability ratio and a low false negative rate in all populations, with a higher positive probability ratio in women than in men. In our population, women were more likely to test positive than men given that most patients in Group 1 were male and most of patients in Group 2 were women. Unlike other studies describing the limited utility of sIgE levels to alpha-gal ≥ 0.35 kUA/L to confirm the diagnosis of mammalian meat allergy in 118 patients showing a high sensitivity (85%) but poor specificity (32%), and including 30 false positives [18], our results illustrate the analysis of internal validity parameters in detail. The differences in sensitivity and specificity compared to other studies [8] are probably due to the use of a lower cut-off point and the comparison with populations from other geographic regions. On the other hand, the analysis of external validity parameters revealed a high negative predictive value (0.98) and global value (0.92), which represented proof of the validity of sIgE levels to alpha-gal for AGS diagnosis in our geographical area.

Some patients in the control group showed a subclinical sensitization to alpha-gal because they tolerated mammalian meat without developing allergic symptoms. The determination of sIgE antibodies can provide essential information on the etiology of the disease because they are produced following exposure of a susceptible individual to an allergen [19], but sensitization is not the same as clinical allergy, and the presence of IgE does not confirm a diagnosis of AGS [20]. The only patient with alpha-gal allergy and sIgE to alpha-gal < 0.1 kUA/L was a woman diagnosed years ago, when she presented with sIgE levels ≥ 0.1 kUA/L, thus being an example of a false negative result. A possible explanation is that sIgE levels alpha-gal tend to decrease over time, except in patients with a history of anaphylaxis, whose levels are maintained [21].

The main limitation of this study was the small sample size, since it made it difficult to carry out comparative statistical analyses between the two groups of patients. Other limiting factors were the heterogeneity of the sample, the lack of analysis of cofactors according to sex, the severity of the reaction, and the time between the performance of the study and the presentation of the reaction since they could influence the final diagnosis. Nevertheless, to our knowledge, this is the first detailed analysis on sIgE to alpha-gal adjusted by sex as a diagnostic tool in AGS.

In conclusion, we described the diagnostic validity of sIgE levels to alpha-gal in AGS in our population. In accordance with these results, sIgE to alpha-gal is a useful diagnostic tool, although it requires further evaluation to explore its correlation with other factors, other available diagnostic techniques or other potential diagnostic tools.

## Data Availability

The datasets generated and/or analyzed during the current study are not publicly available because they are stored at site file investigator but are available from the corresponding author on reasonable request.

## Abbreviations

AGS: Alpha-gal Syndrome
sIgE: Specific IgE
SPT: Skin prick test
ISM: Indolent systemic mastocytosis
BAT: Basophil activation
MAT: Mast cell activation test
HR: Histamine-release
kUA/L: Kilounits or antibody per liter
